# Supplementary material for: Biological Characteristics and Osteogenic Differentiation of Ovine Bone Marrow Derived Mesenchymal Stem Cells Stimulated with FGF-2 and BMP-2
Source: Int J Mol Sci. 2020 Dec 20;21(24):9726. doi: 10.3390/ijms21249726 (PMC7766718; doi:10.3390/ijms21249726)
Supplement: Supplementary file 1 [file ijms-21-09726-s001.pdf]

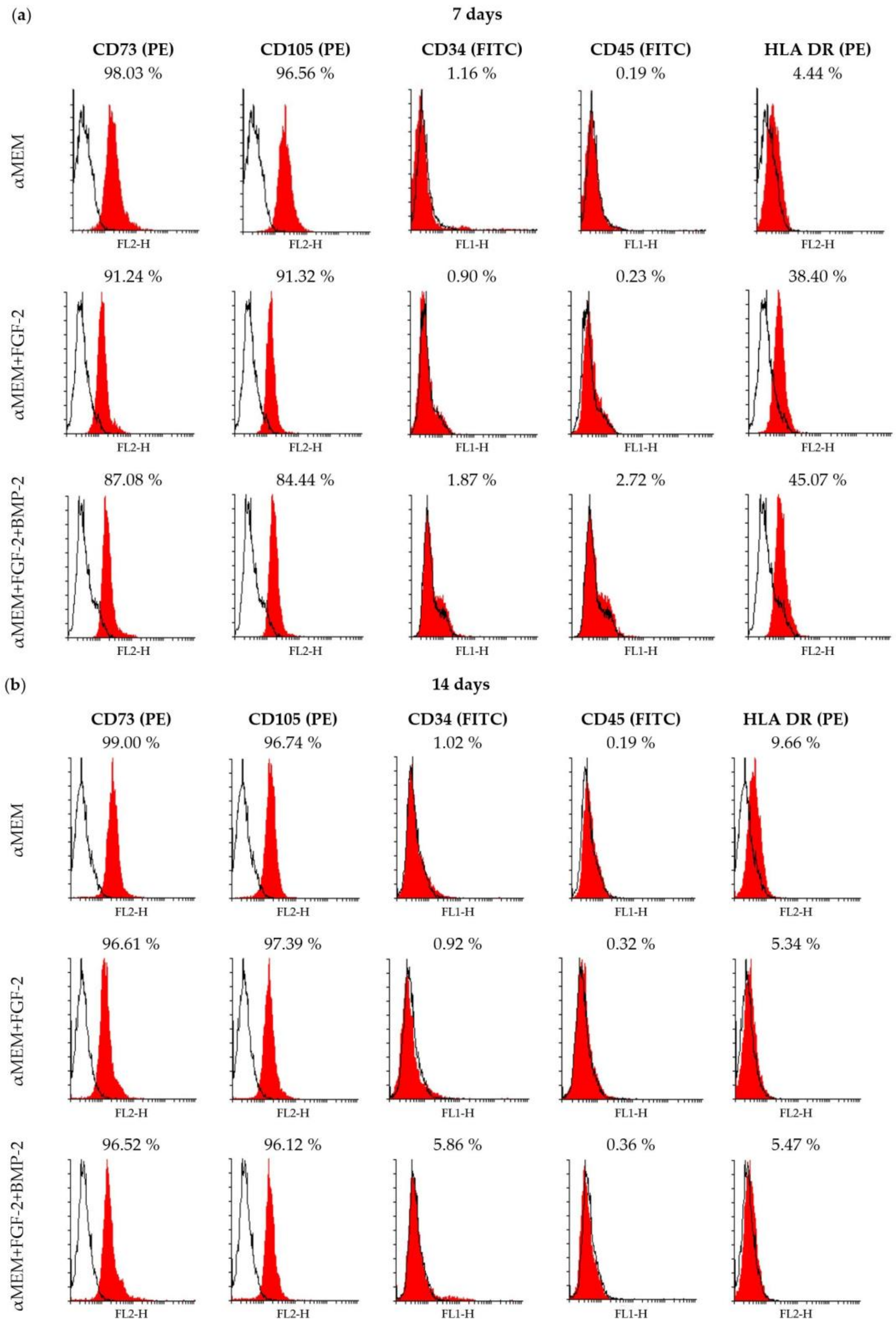

Figure S1. Flow cytometry analysis of BM-MSCs cultured in the control medium αMEM and in a medium supplemented with BMP-2 and/or FGF-2 for 7 and 14 days (**a**, **b**). Cell surface markers of BM-MSCs are similar in all culture conditions and incubation times.

Table S1. Antibodies used for flow cytometry and immunofluorescence.

Antigen	Antibody clone	Company	Dilution
**Direct flow cytometry**			
**CD 73 PE**	TY/23	BD Pharmingen, California, USA	1:5
**CD 105 PE**	MJ7/18	BD Pharmingen, California, USA	1:5
**HLA-DR PE**	37.68	Novus Biologicals, Abingdon, UK	1:250
**Isotype control PE**		Abcam, Cambridge, UK	1:50
**Indirect flow cytometry**			
**CD34**	EP373Y	Abcam, Cambridge, UK	1:250
**CD45**	CACTB51A	Novus Biologicals, Abingdon, UK	1:250
**Goat Anti-Rabbit FITC**		Abcam, Cambridge, UK	1:500
**Goat Anti-Mouse FITC**		Abcam, Cambridge, UK	1:500
**Immunofluorescence staining**			
**Anti-Collagen I**	Rabbit polyclonal	Abcam, Cambridge, UK	1:100
**Anti-Osteocalcin**	OCG3	Abcam, Cambridge, UK	1:100

Table S2. Primers used in PCR assays.

Gene	Primer Sequences (5′-3′)	Fragment size (bp)	Cycles	Tm (°C)
**RT-PCR assay**				
GAPDH	F: CCTGCACCACCAACTGCTTG<br>R: TTGAGCTCAGGGATGACCTTG	224	30	55
CD90	F: AGGACACAGGAAGCCACAAG<br>R: CCCTCACTCTCCATCAGGTC	311	30	56
**qRT-PCR assay**				
GAPDH	F: GCAAGTTCCACGGCACAG<br>R: GGTTCACGCCCATCACAA	249	40	58
BMP-2	F: ATGGTTTCGTGGTGGAGGTAG<br>R: ACTTGAGGCGTTTCCGCTGTT	210	40	58
Runx2	F: TCGCCTCACAAACAACCA<br>R: AGGGACCTGCGGAGATTA	102	40	53
Osterix	F: CAGCGGCGTGCAGTAAAT<br>R: CTGGGAACGAGTGGGAAAA	240	40	56
Collagen type I	F: CAAGAAGAAGACATCCCACC<br>R: AGATCACGTCATCGCACA	133	40	55
Osteocalcin	F: AGATGCAAAGCCTGGTGATGC<br>R: CTCCTGGAAGCCGATGTGGT	211	40	60
Osteopontin	F: TCCCACTGACATTCCAACAA<br>R: CTGTGGCATCTGGACTCTCA		40	60
